# Optogenetically Engineered Neurons Differentiated from Human SH-SY5Y Cells Survived and Expressed ChR2 in 3D Hydrogel

**DOI:** 10.3390/biomedicines10071534

**Published:** 2022-06-28

**Authors:** Si-Yuen Lee, Julian George, David Nagel, Hua Ye, Leonard Seymour

**Affiliations:** 1 Department of Oncology, University of Oxford, Old Road Campus Research Building, Roosevelt Drive, Oxford OX3 7DQ, UK; 2 Institute of Biomedical Engineering, University of Oxford, Old Road Campus Research Building, Roosevelt Drive, Oxford OX3 7DQ, UK; julianhsg@gmail.com (J.G.); hua.ye@eng.ox.ac.uk (H.Y.); 3 Aston Research Centre for Healthy Ageing, Life and Health Sciences, University of Aston, Birmingham B4 7ET, UK; d.nagel@aston.ac.uk

**Keywords:** optogenetics, channelrhodopsin-2 (ChR2), neuronal differentiation, SH-SY5Y cells, RGD-alginate, hydrogels, 3D culture, neurodegenerative disease

## Abstract

The cases of brain degenerative disease will rise as the human population ages. Current treatments have a transient effect and lack an investigative system that is physiologically relevant for testing. There is evidence suggesting optogenetic stimulation is a potential strategy; however, an in vitro disease and optogenetic model requires a three-dimensional microenvironment. Alginate is a promising material for tissue and optogenetic engineering. Although it is bioinert, alginate hydrogel is transparent and therefore allows optical penetration for stimulation. In this study, alginate was functionalized with arginine-glycine-aspartate acid (RGD) to serve as a 3D platform for encapsulation of human SH-SY5Y cells, which were optogenetically modified and characterized. The RGD-alginate hydrogels were tested for swelling and degradation. Prior to encapsulation, the cells were assessed for neuronal expression and optical-stimulation response. The results showed that RGD-alginate possessed a consistent swelling ratio of 18% on day 7, and degradation remained between 3.7–5% throughout 14 days. Optogenetically modified SH-SY5Y cells were highly viable (>85%) after lentiviral transduction and neuronal differentiation. The cells demonstrated properties of functional neurons, developing beta III tubulin (TuJ1)-positive long neurites, forming neural networks, and expressing vGlut2. Action potentials were produced upon optical stimulation. The neurons derived from human SH-SY5Y cells were successfully genetically modified and encapsulated; they survived and expressed ChR2 in an RGD-alginate hydrogel system.

## 1. Introduction

The invention of new treatments and drugs for neurodegenerative diseases remains a main challenge for researchers due to the limited approaches available, which usually provide symptomatic relief to the patients. One of the new strategies, optogenetic stimulation, shows its potential as an alternative to conventional electrical stimulation; however, not many studies have been conducted in vitro without sacrificing animals [1,2]. Although transgenic animal models were commonly used in understanding the mechanism and pathological pathways, they could not completely imitate the human neurodegeneration and intrinsic microenvironment. The lack of realistic in vitro cellular models of such diseases and optogenetic application leads to the development of three-dimensional (3D) investigative systems which involve biomimetic materials. 

Among the natural and synthetic polymer materials under investigation for optogenetic application and neuronal differentiation, alginate displays many desired features, such as having excellent encapsulation efficiency, high water content, biocompatibility, and moldability, and it is easily modified to recapitulate the extracellular matrix (ECM) [3,4]. Alginate is a natural anionic polysaccharide extracted from brown algae and a linear polymer, consisting of α-l-guluronic acid (G) and (1–4)-linked β-d-mannuronic acid (M) monomers [5,6]. Based on the sequence of G and M units, there are three structures formed e.g., M-, G-, and MG-sequential blocks [6]. The synthesis of alginate hydrogels involves chemical or physical crosslinking. Divalent cations, including barium (Ba^2+^), calcium (Ca^2+^), magnesium (Mn^2+^), strontium (Sr^2+^), zinc (Zn^2+^) or copper (Cu^2+^) are commonly used crosslinkers to form ionic bridges between G units [3]. The concentration of cation and exposure time are key factors in tuning the physical and mechanical properties of alginate hydrogels [7]. In the current research, alginate is used to model neural tissue, as reported by Bordoni and colleagues, to differentiate SH-SY5Y cells seeded on a conductive scaffold made of alginate and to show how electrical conductivity promoted the formation of a neural network [8].

Functionalized alginate hydrogels with RGD peptides stimulate integrin-mediated cell adhesion [9,10]. RGD-alginate has been utilized to construct 3D microenvironments for various cell types as well as to regulate and enhance different aspects of the behavior of mesenchymal stem cells (MSC) [11], endothelial cells (EC) [12], induced pluripotent stem cells (iPSC) and neurons [13], among others, when compared to the correspondent non-modified polymers. In the previous study, we fabricated a 3D cellular system using combinations of alginate and RGD with the incorporation of neuronal-specific protein during the encapsulation and culture of stem cell-derived neurons [12]. In the present study, the RGD-alginate hydrogels were further characterized for their physical properties, cell growth, and opsin expression to serve as a potential model of neurodegenerative disease. Investigation on the capability of these hydrogels in supporting optogenetic application, particularly on neurons derived from the most economic, robust human SH-SY5Y cells in an in vitro 3D microenvironment has not been reported. 

When applied in tissue engineering and regenerative medicine, optogenetics allows precise non-invasive control and functional analysis of neuronal cells cultured in vitro [1,14]. Optogenetics involves using genetic engineering to integrate light-sensitive proteins (opsins), such as the most used channelrhodopsin-2 (ChR2) into the cell genome. The ion channel opens in response to blue light [15,16]. To date, optogenetic applications in neurodegenerative diseases were reported to have induced memory retrieval in transgenic mouse models of early Alzheimer’s disease and improved both hypokinesia and bradykinesia of Parkinson’s disease. All these studies were performed in an in vivo environment [17,18]. Since the introduction of opsins into the membrane of neurons supports noncontact spatiotemporal control of cellular activity with millisecond resolution, this technology also potentially allows functional evaluation of neurons differentiated from SH-SY5Y cells with blue-light stimulation in an in vitro 3D platform. The human SH-SY5Y cell line is stable, susceptible to genetic manipulation, and possesses the ability to undergo rapid large-scale expansion as well as simple yet efficient neuronal differentiation [15,19].

In this study, differentiation-inducing agents, retinoic acid (RA) in combination with neurotrophins, (e.g., brain-derived neurotrophic factor, BDNF) [16,20,21,22] were utilized to direct neuronal differentiation. During differentiation, the cells were optogenetically modified and then stimulated with blue light at 430 nm wavelength. Optogenetic construct for targeting neuronal cells, including transduction patterns of the optogenetic components: pLenti-synapsin1-hChr2-(E123T-T159C)-EYFP-WPRE and pLenti-CaMKII-hChr2 (E123T-T159C)-EYFP-WPRE were assessed. In the RGD-alginate hydrogels, survival of the optogenetically engineered cells and ChR2 expression upon encapsulation was evaluated.

## 2. Materials and Methods

### 2.1. Materials

Ultrapure alginate (Pronova-MVG) was purchased from Pronova Biomedical, Oslo, Norway (Mw = 231 kDa, high guluronic acid content, medium viscosity > 322 cP). Live–dead cell staining kit, peptide arginine-glycine-aspartate acid (RGD), and culture medium were acquired from Sigma-Aldrich (St. Louis, MI, USA).

### 2.2. Synthesis and Modification of Alginate Hydrogels 

Alginate was dissolved in 0.9% (*w*/*v*) sodium chloride (NaCl) at 60 °C for 6 h to produce a solution of alginate at a stock solution of 2.0% (*w*/*v*) and diluted to a series of lower concentrations, (e.g., 1.2%, 1.4%, 1.6% and 1.8%). The alginate solution was transferred to a 5 mL syringe, which was connected to a syringe pump (Harvard Apparatus, Holliston, MA, USA) with a flow rate set at 3 mL/min. To polymerize the solution into hydrogel beads, the solution was extruded into a stirred bath of 102 mM calcium chloride (CaCl_2_) using a 30-gauge needle at a distance of 7 cm from the needle nozzle to the CaCl_2_ bath and gently stirred for 7 min at room temperature (RT). Based on a screening test, the concentration of 1.8% (*w*/*v*) of alginate was selected as a control group in this study. 

RGD-alginate was prepared using the same method as previously reported [10,13]. Purified and lyophilized alginate was fully dissolved in MES buffer (0.1 M, at pH 6.5 and containing 0.3 M NaCl) to make alginate-MES buffer. Then, modification or conjugation was performed using carbodiimide (EDC) chemistry. 1-ethyl-(dimethylaminopropyl) carbodiimide (EDC, 0.2 mmol) and *N*-hydroxy-sulfosuccinimide (Sulfo-NHS, 0.1 mmol) were mixed into the alginate-MES buffer on a stir plate for 30 min. RGD peptide was added and allowed for a 20 h reaction, then purified for 72 h by dialysis (molecular weight-cutoff 3500 Dalton) against distilled water in decreasing salt solution concentrations. The RDG-alginate solution was lyophilized and stored in a desiccator until used. The reaction yield was calculated using amino acid analysis. During cell encapsulation, laminin (20 µg/mL) was added to the RGD-alginate solution prior to polymerization. 

### 2.3. Characterization of RGD-Alginate Hydrogels

#### 2.3.1. Swelling Test

The swelling ratio of RGD-alginate hydrogels was evaluated by swelling in phosphate buffer saline (PBS) at pH 7.4. The initial mass of the unmodified alginate and RGD-alginate hydrogels was recorded on day 0 (W_i_) prior to immersion in 2 mL of PBS for 1, 3, 7, and 14 days at 37 °C. At each time point, the hydrogel beads (per mL) were taken out of the PBS solution. The swollen samples were blotted using filter paper to remove excess PBS on the surface of the hydrogels, and the mass of the samples was measured again (W_s_). The swelling amount (S) of the hydrogels was defined as a ratio of the mass increase (W_s_ − W_i_) divided by the initial weight (W_i_):

S (%) = (W_s_ − W_i_)/W_i_ × 100,

where W_i_ indicates the initial weight at day 0 and W_s_ indicates the swollen weight of the hydrogels at each time point.

#### 2.3.2. Degradability Measurement

The in vitro degradation experiments of alginate hydrogels were conducted by incubating unmodified alginate hydrogels and RGD-alginate hydrogels in PBS (pH 7.4) at 37 °C. The average hydrogel mass at day 0, and average loss of mass at 1, 3, 5, 7, and 14 days were determined by removing the hydrogels from the PBS (per mL with average of 30 hydrogel beads), gently blotting dry the surface of hydrogels with filter paper and freezing the hydrogels at −80 °C prior to lyophilization. Degradation (%) was assessed by measuring the loss of mass, defined by the following equation:

Degradation (%) = (W_i_ − W_d_)/W_i_ × 100,

where W_i_ indicates the initial dry weight at day 0 and W_d_ indicates the dry weight of the hydrogels at each time point. 

### 2.4. Optogenetic Modification and Neuronal Differentiation of Human SH-SY5Y Cells

#### 2.4.1. Lentiviral Production

DNA plasmids coding for ChR2 and its mutants were obtained from Karl Deisseroth, Department of Bioengineering and Howard Hughes Medical Institute, Stanford University, California, USA, for the hChR2 and Peter Hegemann, Institute of Biology, Experimental Biophysics, Humboldt-University, Berlin, Germany, for the ChR2-T159C mutant. These plasmids were integrated into a lentiviral expression vector (gifted by Dr. David Nagel and Dr. Eric Hill, Aston University, UK). The ChR2 gene was fused to yellow fluorescent protein (YFP) and cloned into a lentivirus expression plasmid with cell-type-specific promoter (i) neuron-specific promoter (pLenti-synapsin1-hChr2-(E123T-T159C)-EYFP-WPRE) and (ii) glutametergic neuron promoter (pLenti-CaMKII-hChr2 (E123T-T159C)-EYFP-WPRE). The universal promoter, human elongation factor-1 alpha (EF1a) labeled with green fluorescent protein (GFP), was included in the study. 

Human embryonal kidney 293 FT cell line, HEK 293FT (Invitrogen, Waltham, MA, USA) was used as a host for lentiviral production. The cells were cultured until 80–90% confluent and sub-cultured to a minimum of three passages before transfection. Replication-incompetent lentiviruses were produced via a second-generation packaging system containing the promoter and gene of interest, (e.g., SYN1-ChR2-YFP, CaMKII-ChR2-YFP, and EF1a-ChR2-GFP), the viral helper plasmid (psPAX2), and the pseudotyping plasmid (pMD2.G, encoding the coat protein VSV-G). At 100% confluence, the cells were transfected with DNA using lipofectamine-2000: 10 µg of plasmid containing the promoter and gene of interest, 10 µg of pSPAX2, and 10 µg of pMD2.G were mixed with 75 µL of lipofectamine-2000 and 1.5 mL of Opti-MEM. Approximately 48 h later, the supernatant was harvested, filtered, and stored at −80 °C until use.

#### 2.4.2. Neuronal Differentiation

The human SH-SY5Y cells were seeded in black 24-well glass-bottom plates coated with poly-L-lysine and/or laminin to support neurite outgrowth and differentiation. Cells were cultured for one day before differentiation was initiated by adding 10 μM RA to the neurobasal media. The medium with RA was replaced every day and incubated for up to five days. The cells were further matured in maintenance medium consisting of neurobasal media and DMEM/F-12 supplemented with 0.1% BDNF, 0.5% N-2, and 0.5% B-27 for more than two days before use.

### 2.5. Evaluation of Opsin Gene Expression and Differentiated Neuronal Population

#### 2.5.1. Optogenetic Transduction and Efficiency

The differentiated SH-SY5Y cells were transduced with ChR2-YFP or ChR2-GFP transgenes using lentiviruses containing elongation factor 1-alpha (EF1a, universal) promoter, synapsin-1 (SYN1, pan-neuronal) promoter, and calmodulin-dependent protein kinase type II (CaMKII, glutamatergic neurons) promoter, which had been generated as previously described (refer to section on lentiviral production). The viral load used in the transduction was MOI-2 for the first infection in 24 h and second infection in the next 24 h. Transduction efficiency of differentiated human SH-SY5Y cells was evaluated using fluorescent microscopy and flow cytometry. The cells containing SYN1, CaMKII, and EF1a promoters were trypsinized from the culture at day 14 and 28. Detached cells were washed with culture medium, and immersed in PBS and the level of transduction was quantified by flow cytometry (BD FACSCalibur). Positive gate regions were established using non-transduced cells, which served as a negative control. A total of 10,000 events were acquired for each sample and the data were analyzed with BD CellQuest™ Pro Software (Brea, CA, USA). Cell viability was also measured with trypan blue staining and by a cell count. 

#### 2.5.2. Immunofluorescent Staining for Neuronal Expression

The optogenetically modified SH-SY5Y cells were fixed with 4% paraformaldehyde in PBS and incubated for 30 min at RT. Cells were washed three times with PBS, permeabilized, and blocked (5% BSA, 0.2% Triton-X100, and 0.1% Tween 20 in PBS) for 60 min and incubated overnight in primary antibody solution at 4 °C. Primary antibodies were mouse anti-neuron-specific class III beta-tubulin (Tuj1) (1:1000; Abcam, Cambridge, UK), mouse anti-γ-aminobutyric acid B receptor 1(GABA-B-R1) (1:50; Abcam), rabbit anti-glial fibrillary acidic protein (GFAP) (1:2500; Abcam), and rabbit anti-vesicular glutamate transporter 1 (vGlut1) (1:100; Abcam). Cells were washed three times with wash buffer (0.2% BSA, 0.2% Triton-X100, and 0.1% Tween 20 in PBS) prior to being blocked for 30 min with 10% goat serum in wash buffer. Next, secondary antibody solution was added and the cells were incubated at RT for 2 h. Alexa Fluor^®^568-conjugated goat anti-mouse, Alexa Fluor^®^649-conjugated goat anti-rabbit, and Alexa Fluor^®^488-conjugated goat anti-rabbit secondary antibodies were all used at 1:500 (Invitrogen, Grand Island, NY, USA). Finally, cells were washed three times with washing buffer and 300 µL of 6-diamidino-2-phenylindole (DAPI) was added. Cells were examined using confocal microscopy (Zeiss-LSM 780, Oberkochen, Germany) and ZEN light software (version 2013, Oberkochen, Germany). 

#### 2.5.3. Optogenetic Stimulation and Calcium Imaging

Calcium dye, CAL-590 (Acetoxymethyl (AM), Mw-1129.86, ATT Bioquest) was used to stain the cells for live calcium imaging. Optical stimulation was applied through efficient blue light excitation (470–490 nm) of a confocal microscope equipped with argon lasers (Carl Zeiss 780, Oberkochen, Germany). Transduced and non-transduced differentiated human SH-SY5Y cells were then incubated in the dark with CAL-590 AM (10 µM in artificial cerebrospinal fluid, ACSF) for 40 min at RT. Finally, the cells were washed and incubated in ACSF for confocal microscopy imaging. The setup was referred to in our previous study and described as follows [13]: 20× objective, Zoom 1, 512 × 512 format, 8-bit resolution, xyt scan mode, 400 Hz speed, 1.0 airy pinhole, frame average 1, line average 4 for image, and 1 for time-lapse imaging. The optogenetically engineered and differentiated human SH-SY5Y cells were excited with 568 nm laser for Ca^2+^ indicator (CAL-590 AM) and 488 nm laser (at 20% of laser) to view the yellow fluorescence from YFP whilst 100% of 488 nm laser was set to stimulate the ChR2-YFP. Time-lapse imaging was set with a stimulation of 1.635 s and a total of 200 frames were recorded at the basal Ca^2+^ level recording [13]. 

### 2.6. In Vitro Culture in RGD-Alginate Hydrogel and Evaluation

The differentiated and optogenetically engineered human SH-SY5Y cells were encapsulated in RGD-alginate hydrogel and cultured with maintenance medium for up to 14 days. The constructs were withdrawn from the 3D culture for experiments and analyses at certain time points. 

#### 2.6.1. Cell Viability

Cells at 2 × 10^6^/mL were added into the RGD-alginate solution for encapsulation and polymerization to form an average of 30 hydrogel beads. The constructs were assessed with live–dead cell staining for cell viability testing (Calcein-AM, Sigma-Aldrich, St. Louis, MI, USA). A series of z-stack images were captured using fluorescent microscopy (Nikon T_i_ Eclipse, Tokyo, Japan), and processed with IMARIS software (version 8, Bitplane, Belfast, UK) to show the distribution and localization of live and dead cells. 

#### 2.6.2. ChR2 Expression

Upon cell encapsulation with RGD-alginate hydrogels, the optogenetically modified and unmodified cells (control) were viewed with a confocal microscope (Zeiss-LSM 780, Oberkochen, Germany) to examine the expression of ChR2-YFP, which contained CaMKII promoter (labeled with green fluorescence). 

### 2.7. Statistical Analysis

All values are presented with means ± standard of deviation (SD), except where otherwise indicated. The differential significance of the results obtained was tested by two-way analysis of variance (ANOVA), using SPSS software (SPSS Inc., Chicago, IL, USA). The *p*-value is denoted as * = *p* < 0.05, *** = *p* < 0.001, n.s. = non-significant. Experiments were performed in triplicate (*n*) and at least three independence tests (N).

## 3. Results and Discussion

### 3.1. Physical Properties of 3D Hydrogels

**Figure 1:**
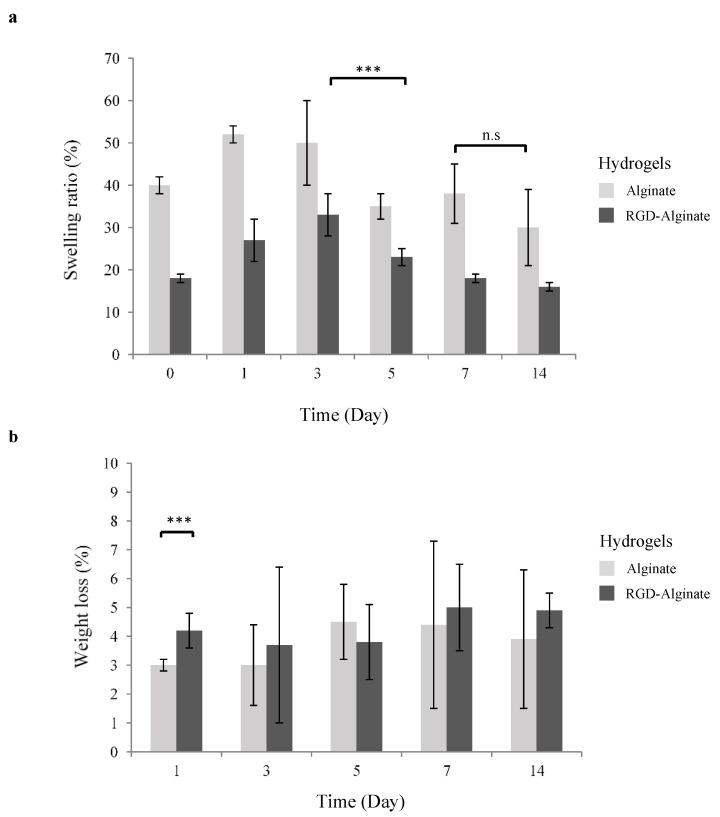
Physical properties of RGD-alginate hydrogel as compared to unmodified alginate hydrogel. (**a**) Swelling profile and (**b**) in vitro degradation profile. The hydrogels were incubated in PBS pH 7.4 at 37 °C, removed from the incubation at different time points and data were measured (*n* = 3). ANOVA test was used to determine significance; n.s = non-significant; *** = *p* < 0.001 was considered statistically significant.

The swelling and degradation profiles of RGD-alginate hydrogels are important criteria to consider in clinical translation. Hydrogels provide adherence and proliferation sites for cell culture in a 3D microenvironment. Their rate of swelling and degradation should match the growth of cells and application in optogenetics. To assess the swelling properties of alginate hydrogels, the change in hydrogel weight during incubation under physiological conditions (in a PBS solution, pH 7.4 at 37 °C) was measured. As observed, alginate hydrogels showed a higher swelling ratio than RGD-alginate, both undergoing a weight increase at the beginning before decreasing after day 3 (Figure 1a). This behavior was anticipated as hydrogels are able to swell in aqueous solutions [23,24]. Greater osmotic pressure from the PBS into the hydrogels was created when the hydrogels were transferred to PBS incubation [23,25]. The hydrogel volume subsequently expanded as the void regions of the polymer network filled with PBS until an equilibrium state was reached. The increased swelling may enhance cellular viability within the hydrogels as well as facilitate mass transfer, waste, and oxygen exchange via enlarged pores in the hydrogels. It is noticeable that RGD-alginate hydrogels possessed a similar and constant swelling trend when compared to alginate; the swelling ratio increased until day 3, then decreased constantly and seemed to reach an equilibrium at day 14.

The degradation of alginate hydrogels is mediated by calcium ion exchange in the local environment and not by the cleavage of molecular bonds [26]. The degradation rate of alginate hydrogels is dependent on the physiologic pH of the surrounding medium and the initial ionic strength of the solution used for crosslinking. It has been demonstrated that alginate hydrogels crosslinked at pH 6 or 7 can more stably maintain a swelling ratio than those crosslinked at pH 8 [27]. Likewise, both alginate and RGD-alginate hydrogels crosslinked at pH 7 and incubated in a solution that approximated in vivo conditions in this study, were found susceptible to excessive swelling throughout the 14 days of investigation. The RGD-alginate hydrogels showed low weight loss (4.3%) on day 1 whilst retaining the degradation rate consistently onwards without significant difference (Figure 1b). A similar trend was shown by unmodified alginate hydrogels with a significant difference on day 1. It has been reported that chemical crosslinking method contributed to the stable structure of RGD-alginate hydrogels to remain a low and consistent degradation [28].

### 3.2. Expression of ChR2-YFP in Differentiated Human SH-SY5Y Cells

**Figure 2:**
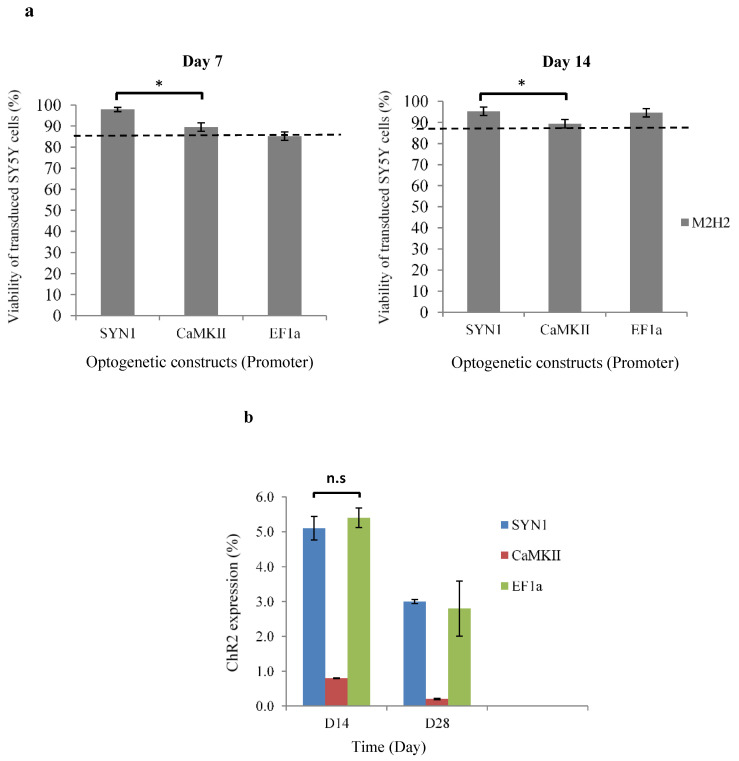

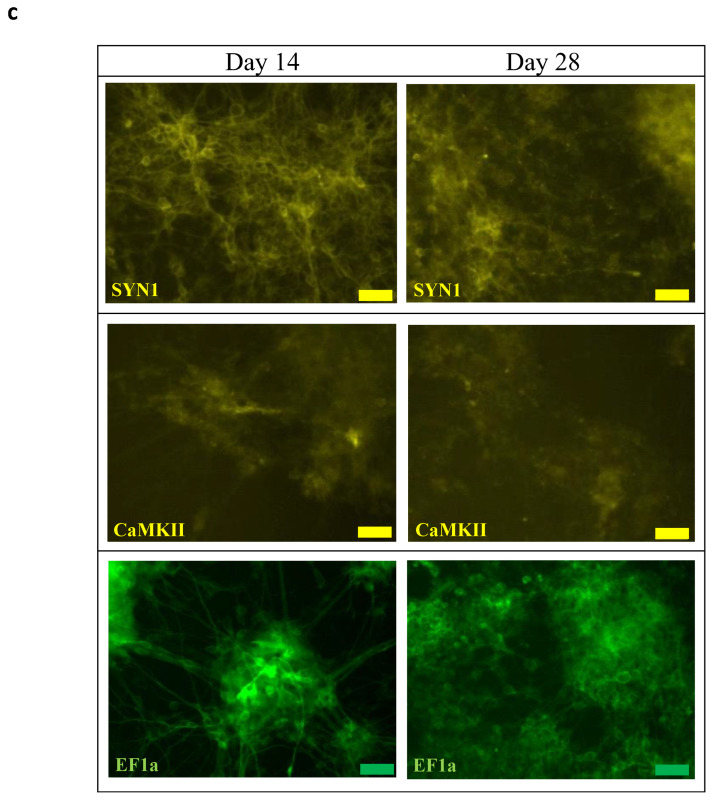
Upon optogenetic modification, neuronal differentiated human SH-SY5Y cells that contained neuron-specific promoters, (e.g., SYN1, CaMKII, EFIa) showed a high percentage of viability (>85%), and positive expression of ChR2. (**a**) Cell viability of lentiviral transduced human SH-SY5Y cells under the condition of MOI-2 was analyzed using a trypan blue exclusion test after 7 and 14 days of transduction (*n* = 3). (**b**) Efficiency of transduction was evaluated using flow cytometry on days 14 and 28 post-transduction (*n* = 3). ANOVA test was used to determine significance; n.s = non-significant; * = *p* < 0.05 was considered statistically significant. (**c**) Optogenetic constructs, pSNY1-ChR2-YFP and pEF1a-ChR2-GFP were strongly expressed in neuronal differentiated human SH-SY5Y cells; however, pCaMKII-ChR2-YFP showed lower expression. Fluorescence imaging of cells was conducted using fluorescence microscopy (Nikon Eclipse T*_i_*-E, Tokyo, Japan). Scale bar: 50 µm.

The ability to confer optogenetic control over different neuron subtypes is dependent upon the control of ChR2 with cell type-specific promoters, such as the use of CaMKII to target excitatory glutamatergic neurons. In this study, lentiviral vectors successfully delivered transgenes ChR2-YFP or ChR2-GFP into the neuronal differentiated human SH-SY5Y cells. ChR2 expression was mediated and driven by CaMKII promoters, including SYN1 and EFIa promoters. An in vitro optogenetically control neurodegenerative disease model was developed using RA-BDNF differentiated human SH-SY5Y cells, and the efficiency of optogenetic transduction was evaluated. Transduction of differentiated human SH-SY5Y cells with viral load at MOI-2 in CaMKII, SYN1, and EFIa-driven cells showed an average of high cell viability (>85%) for 7 and 14 days (Figure 2a). SYN1-driven cells not only had significantly higher viability than CaMKII but also presented a higher percentage of ChR2 positive cells (Figure 2b) and a stronger expression level (Figure 2c). Higher transduction efficiencies are commonly obtained by increasing viral load or concentration but result in higher toxicity [29]. Interestingly, transduction at MOI-2 followed by re-transduction contributed to high cell viability and positive expression of ChR2. The high viability and low cytotoxicity after lentiviral transduction may be attributed to the human SH-SY5Y cells’ robustness and rapid recovery from cell stress. 

Flow cytometry results revealed that the ChR2 expression level in cells driven by pCaMKII-ChR2-YFP was lower than pSYN1-ChR2-YFP and pEF1a-ChR2-GFP on 14 and 28 days of transduction (Figure 2b). The low expression level and reducing the signal of pCaMKII-ChR2-YFP were further confirmed by the images captured using confocal microscopy (Figure 2c), suggesting that CaMKII is a weaker cell type-specific promoter than SYN1 and EF1a, in line with other reports in in vitro and in vivo studies performed by Dittgen, T. el al., and Rein, M.L. and Deussing, J.M. [30,31]. ChR2-YFP and ChR2-GFP were localized and expressed in the cell membrane and distributed along neurites. Neuron-specific and weaker promoters need to be considered because overproduced transgene products have been reported to be toxic in certain types of cells; thus, optimal degrees and patterns of transgene expression are required [32]. In contrast, a recent study reported positive ChR2 expression in targeting astrocytes using mCherry promoter and adeno-associated viral (AAV) vectors. An optogenetic construct, AAV-GFAP-ChR2(H134R)-mCherry was characterized and resulted in high astrocytic transduction (approximately 88%) with AAV8, successfully establishing light-induced intervention of astrocyte activity in the rat brain cortex [33]. 

### 3.3. Neuronal Expression Post Optogenetically Modification and Differentiation 

**Figure 3:**
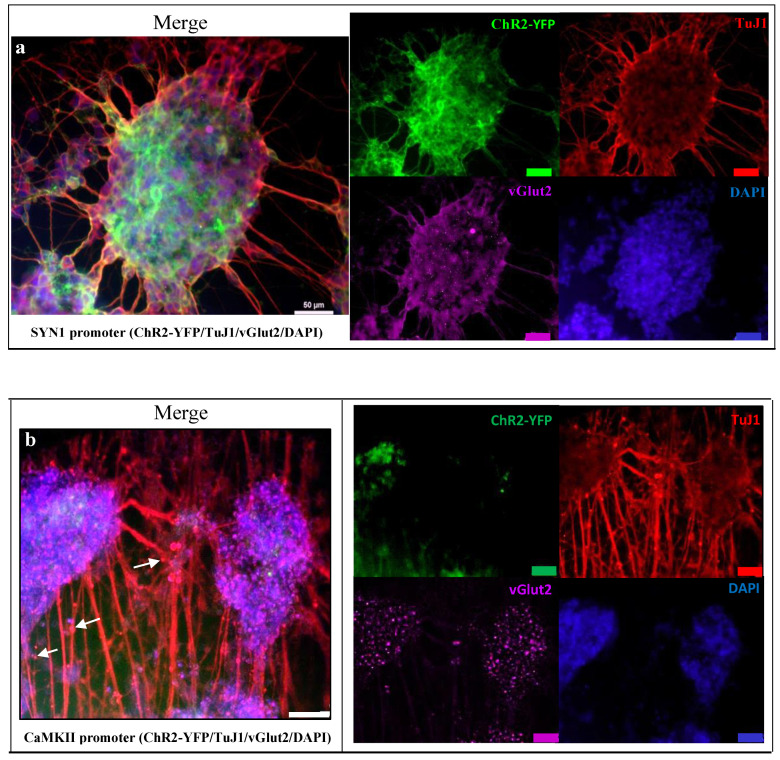
Immunofluorescent staining of neuronal differentiated human SH-SY5Y cells demonstrated positive expression of TuJ1 and vGlut2, containing ChR2-YFP under the control of (**a**) SYN1 promoter and (**b**) CaMKII promoter. The cells were cultured on a laminin-coated 24-well, glass-bottom plate in the complete medium after differentiation. The cells were then fixed, permeabilized, and stained with neural markers, TuJ1 (red) and vGlut2 (magenta) to identify the neuronal population. Opsin gene ChR2-YFP was expressed (labeled with green) whilst DAPI stained the nuclei blue. Functional vesicle transportation appeared as red dots along the neurites as shown in figure b (white arrows). Fluorescence images were obtained using confocal microscopy (Zeiss-LSM 780, Oberkochen, Germany) and processed with ZEN light software. Scale bar: 50 µm.

The neuronal differentiated SH-SY5Y-ChR2-YFP cells expressed mature neuronal markers, and ChR2-YFP was optically excitable in culture. After the sequential treatment with RA and BDNF, the cells exhibited beta III tubulins (TuJ1)-positive long neurites that formed networks characteristic of neurons (Figure 3a,b). The differentiated SH-SY5Y-ChR2-YFP cells exhibited positive staining of TuJ1 and vGlut2. The TuJ1-positive cells displayed typical neural branching and normal nuclei, suggesting that SH-SY5Y-ChR2-YFP cells are non-degenerated after transduction. Functional vesicle transportation was clearly seen along the neurites (Figure 3b). The differences in the level of expression for TuJ1 and VGlut2 may attribute to different cell densities and maturity after a period of neuronal differentiation, genetic modification, and cultures. The cells were formed in layers and aggregates. 

In the differentiated human SH-SY5Y cells, the number of mature neurons, homogeneity, and their functional subpopulation were dependent on the method of differentiation. For instance, sequential treatment of the human SH-SY5Y cells with RA and BDNF has been conducted by other researchers and reported to generate fully differentiated human neuron-like cells or mature neurons. Neurite outgrowth was induced, and neural networks were formed in the culture. The differentiated cells enter apoptotic cell death when BDNF is eliminated from the culture medium [22]. BDNF and NGF are found to react on specific tropomyosin receptor kinases, which activate intracellular kinase signaling cascades that have synergistic effects on downstream effectors to promote the expression of neuro-specific proteins after differentiation [2].

### 3.4. Effects of Optogenetic Stimulation

**Figure 4:**
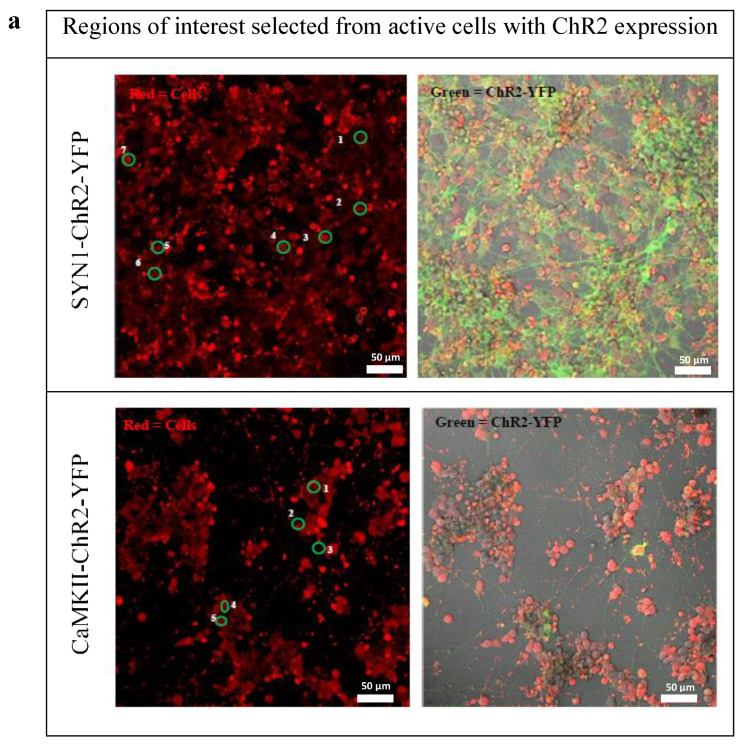

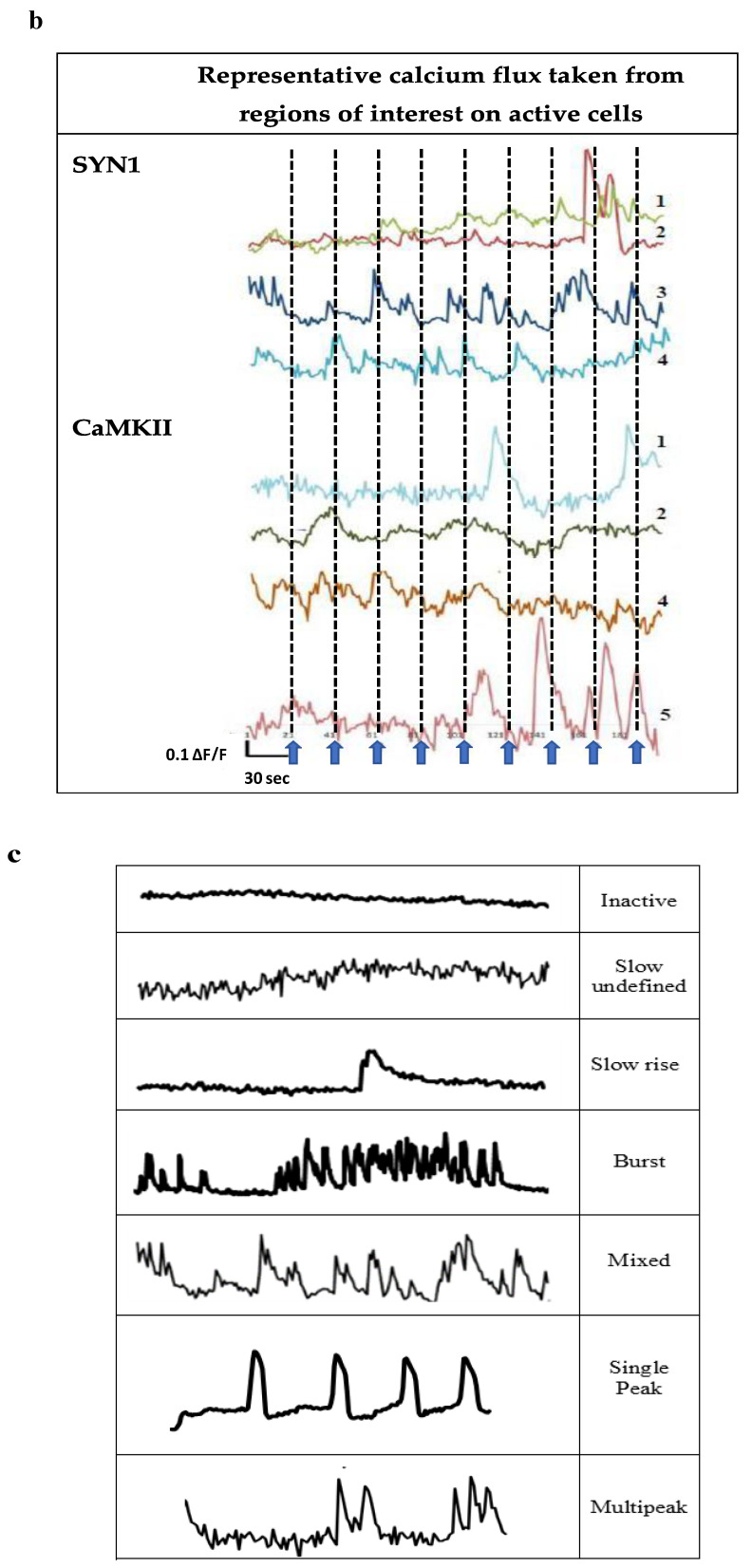
The neurons differentiated from human SH-SY5Y cells expressing ChR2-YFP, which were driven by SYN1 and CaMKII are responsive to optical stimulation. (**a**) The cells were stained with calcium dye (red) and the region of interest (ROI) or selected single cells were labeled with number (left). pSYN1-ChR2-YFP and pCaMKII-ChR2-YFP were expressed (labeled with green) in the culture and the locations were shown in a merged image of bright field and fluorescence micrograph (right). (**b**) The cells were selected from the ROIs as marked in (a) and stimulated with laser at 488 nm every 30 s (200 frames), the fluorescence intensity was normalized to the level of baseline fluorescence measured before the onset of the calcium signal (∆F/F). Small arrow: optical stimulation; circle: region of interest (ROI). Scale bar: 50 µm. (**c**) The traces displayed typical examples of calcium imaging time series over 5 min from different ROIs, which were classified based on the calcium events. Categories of ROIs were distinguished according to: (1) calcium waves such as inactive, slow undefined, slow rise, burst, and mixed waves, and (2) calcium spikes, including single peak and multipeak.

The cells stained with calcium dye were selected from a region of interest (ROI) as represented in Figure 4a. ChR2-YFP expression driven by SYN1 and CaMKII promoter showed synchronous calcium firing in mixed waves consisting of both single and multipeak spikes upon optical stimulation (Figure 4b). Slow rise waves may signify the presence of premature neurons. Results demonstrated that neurons differentiated from human SH-SY5Y cells containing optogenetic constructs, pSYN1-ChR2-YFP, and pCaMKII-ChR2-YFP are both responsive to optical stimulation. Interestingly, although CaMKII is a weaker promoter in this study with lower ChR2-YFP expression detected, action potentials (AP) were triggered upon optical stimulation.

In comparison to other studies, SYN1 promoter-directed ChR2 expression in human embryonic stem cell-derived neurons with various neurotransmitter phenotypes has been reported [34]. Optogenetic stimulation of these cells could reliably trigger AP frequencies of 5–30 Hz depending on cell maturity whilst post-synaptic currents were induced in neurons both in vitro and in vivo (within transplanted tissue) for at least six months [34]. This agreed with the findings in the present study that cell-type-specific targeting of glutamatergic neurons using CaMKII promoter is correlated to the maturity of neurons and their quantity in the culture, subsequently affecting optogenetic control of neural network activities.

### 3.5. Cell Viability and ChR2 Expression in RGD-Alginate Hydrogels

**Figure 5:**
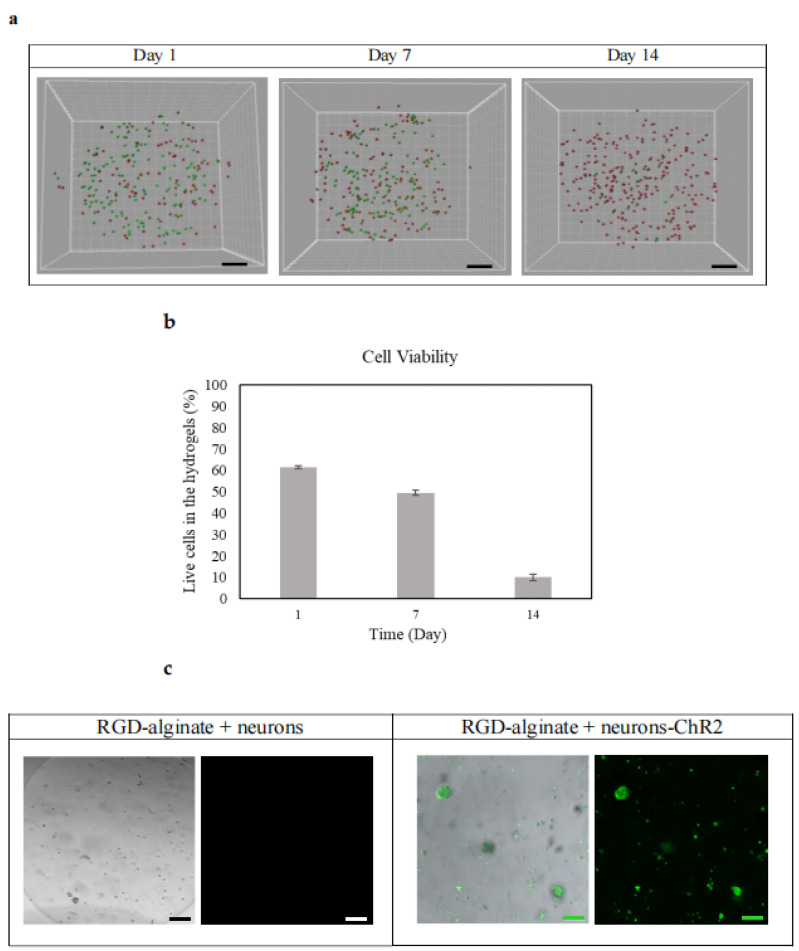
Cell viability and localization of optogenetically engineered neurons encapsulated in the RGD-alginate hydrogels and expression of ChR2 in the hydrogels from day 1 to day 14. (**a**) Live–dead cell staining (Calcein-AM, Sigma-Aldrich, St. Louis, MI, USA) was performed. A series of z-stack images were captured using fluorescence microscopy (Nikon T_i_ Eclipse, Tokyo, Japan) and processed with IMARIS software (version 8, Bitplane, Belfast, UK). Live cells were stained green whilst dead cells were stained red. Scale bar: 200 µm. (**b**) Quantitative analysis of live–dead cells (*n* = 3). (**c**) After 24 h of encapsulation, the cells encapsulated with RGD-alginate hydrogels were investigated using a confocal microscope (Zeiss-LSM 780, Oberkochen, Germany) to evaluate the expression of ChR2 (labeled in green) as well as the morphology and distribution of cells within the 3D culture system. Non-transduced or unmodified cells were used as a control. Scale bar: 100 µm.

Neurons expressing ChR2 showed high cell viability in RGD-alginate hydrogels on days 1 and 7; however, live cells were decreasing when they reached day 14 of culture (Figure 5a,b). The cell death that occurred after 7 days may be due to consistent degradation of the RGD-alginate hydrogels that lead to cell loss or cell detachment. Another possible reason is the density of conjugated RGD that may be insufficient to support the long-term culture of this cell type, which is a limitation of this study. In response to the hydrogel materials used, different cell types and cell densities as well as adhesive peptide/RGD density attribute to different cell–matrix interactions [35]. In addition, the majority of the studies have demonstrated cell viability assessment for up to 7 days only [11]. For example, Raquel Maia and colleagues reported that a low cell seeding density of below 15 × 10^6^ cells/mL, (e.g., 2 × 10^6^, 5 × 10^6^, 7.5 × 10^6^ cells/mL) also exhibited a decreasing cell viability trend in human mesenchymal stem cells when cultured for 1 week in RGD-alginate 3D matrices [11,35]. The concentration of the hydrogel polymer is another key factor. Results showed by Dumbleton and colleagues suggested that the modification of alginate concentration with the RGD peptide resulted in significant changes in the cell morphology [36]. All cell types tested by them were reported to proliferate and grow better on the 2% alginate-RGD hydrogels than 2% alginate without RGD or 0.5% alginate-RGD hydrogels [36]. Live–dead cell staining and IMARIS software further revealed the localization of live/dead cells within the 3D hydrogels, with both live and dead cells distributed evenly (Figure 5b). When the modified and differentiated cells were encapsulated and transferred to 3D culture using RGD-alginate hydrogels, ChR2 was also found to stably express (YFP labeled with green) without being affected by the 3D culture environment and encapsulation process. The cells appeared in spherical aggregates within the RGD-alginate hydrogels upon encapsulation (Figure 5c). The observation is in line with our previous study utilizing human pluripotent stem cell-derived neurons, which were optogenetically modified [13]. When another cell type was used and tested in this study, the results further confirmed the efficiency of optogenetic constructs and demonstrated initial success in the development of a 3D in vitro model of neurodegenerative disease, which responded to optogenetic stimulation. 

The ChR2 neurons derived from differentiated SH-SY5Y cells were expected to respond to optical stimulation and maintained their functionality within RGD-alginate hydrogels for up to 7 days but may affect by increasing cell death from day 7–14 and onwards. To confirm this, future work would be to precisely stimulate and record electrophysiologic activities from the cells in RGD-alginate hydrogels at different time points. The use of advanced optical tools, such as lasers to stimulate specific cells or selected regions with different wavelengths, and the efficiency of light penetration into the hydrogels should be investigated [37,38]. In order to prolong cell survival post 7 days of encapsulation and long-term culture in 3D, the RGD-alginate hydrogels required further modification and improvement, including increasing their porosity and peptide density, adding the conducive component(s), combining the other polymer(s), and applying advanced fabrication technology (e.g., bioprinting). Other researchers utilized alternative approaches to introduce the RGD sequence by combining collagen or gelatin with alginate hydrogel as an in vitro study system [39,40,41]. In addition, mechanical characterization of the improved hydrogels is suggested to be included in the future study. 

## 4. Conclusions

In this study, we combined the pioneering optogenetics and 3D culture technology using hydrogel to establish a light-responsive in vitro human neurodegenerative disease model. The encapsulated cells derived from human SH-SY5Y cells were successfully optogenetically modified and differentiated to mature neurons. These cells can serve as an alternative cell source for optogenetic application in a 3D microenvironment apart from human stem cell-derived neurons, which have been explored and reported in the previous study. The RGD-alginate hydrogels supported cell viability for up to 7 days and the cells expressed ChR2 upon encapsulation. The transduction efficiency and cell survival rate were high; however, optogenetic construct pLenti-CaMKII-hChr2 (E123T-T159C)-EYFP-WPRE demonstrated a lower expression of ChR2 in targeting specific-neuronal cells. In addition, RGD-alginate hydrogels had a lower swelling property than non-functionalized alginate hydrogels. Their consistent degradation rates helped to prevent rapid disintegration, making them suitable for cell culture. However, further modification and improvement of the hydrogels are required for long-term culture, especially to support more than 2 weeks of cell cultivation. In summary, the 3D hydrogel system showed some excellent properties to be used in developing a future optical control and responsive in vitro model of neurodegenerative disease. Here, we have also provided a foundation for the characterization of the optogenetic constructs for the light-induced intervention of specific neuronal cell activity in 3D hydrogels. 

## Data Availability

Not applicable.
